# Polyaniline/Ag nanoparticles/graphene oxide nanocomposite fluorescent sensor for recognition of chromium (VI) ions

**DOI:** 10.1038/s41598-020-70678-8

**Published:** 2020-08-12

**Authors:** Shaker Ebrahim, Azza Shokry, M. M. A. Khalil, Hesham Ibrahim, Moataz Soliman

**Affiliations:** 1 Department of Materials Science, Institute of Graduate Studies and Research, Alexandria University, P.O. Box 832, Alexandria, Egypt; 2 Department of Environmental Studies, Institute of Graduate Studies and Research, Alexandria University, P.O. Box 832, Alexandria, Egypt; 3 Department of Nanotechnology and Composite Materials, Institute of New Materials and Advanced Technology, City of Scientific Research and Technological Applications (SRTA- City), New Borg El Arab City, P.O. Box 21934, Alexandria, Egypt

**Keywords:** Environmental sciences, Chemistry, Materials science, Nanoscience and technology, Optics and photonics

## Abstract

Selective determination of toxic hexavalent chromium (Cr(VI)) is a stringent important due to its huge negative impact on the health and environment. Recently, the high sensitivity, rapidness, and cost-effectiveness of the fluorescent sensors for Cr(VI) have been developed. A fluorescent nanocomposite (NC) has been synthesized based on doped polyaniline (PANI), 2-acrylamido-2-methylpropanesulfonic acid (AMPSA) capped Ag nanoparticles (NPs) and graphene oxide (GO) quantum dots (QDs) via in situ reaction for highly selective sensing of Cr(VI) ions based on the luminescent quenching in the range from 0.01 to 7.5 mg/L. This NC showed an emission peak at 348 nm with a linear range from 0.01 to 0.05 mg/L and the low limit of detection (LOD) was 0.0065 mg/L (~ 6 µg/L). PANI/Ag (AMPSA) GO QDs NC displayed high selectivity for Cr(VI) over other common metal ions. Notably, the PANI/Ag (AMPSA) GO QDs NC can be used for distinguishing Cr(VI) and Cr(III) in solutions. The sensitive determination of Cr(VI) in real surface water samples was also confirmed and demonstrated recoveries in the range 95.3–99.2%. This NC will emerge as a new class of fluorescence materials that could be suitable for practical applications.

## Introduction

Monitoring and detection of toxic heavy metal ions are in urgent demand due to their potential highly carcinogenicity mutagenicity^1^. In the aquatic environments, chromium exists as Cr(III) and Cr(VI) oxidation states. Cr(VI) is very harmful due to its high carcinogenic and mutagenic properties while Cr(III) is harmless and safe^2^. The permissible limit of Cr(VI) in drinking water was set as 50 μg/L according to the World Health Organization (WHO) report^3^. Therefore, the ability of selectively detection of Cr(VI) without the interference of Cr(III) is very important for the human health and environment impact assessments. There are different techniques commonly used for the detection and sensing of Cr such as the ion chromatography (IC)^4^, inductively coupled plasma mass spectrometry (ICP-MS)^5^ and atomic absorption–emission spectroscopy^6,7^. Eaton et al. have revealed significant ambivalences between the results obtained by these techniques^8^. They analyzed the concentrations of both Cr(VI) and total Cr over 1,500 water samples. They found that in almost half of analyzed samples, the determined Cr(VI) concentrations by the IC were higher than the total Cr concentrations determined by ICP-MS which indicate deficiencies in the discrimination between Cr(VI) and Cr(III) ions of these commonly used methods. Therefore, there is a growing demand to develop reliable analytical techniques for selective and accurate Cr(VI) determination.

The new fluorescent technique presents different advantages such as high sensitivity, fast response time, visualization and high selectivity^9^. High luminescent advanced materials such as porous organic polymers, nanoparticles and carbon dots have been explored to sense Cr(VI)^10–12^. Although good performance has been obtained, these advanced materials suffered from low sensitivity and low stability^9^. So, the potential materials with excellent stability for sensitive detection and highly selective of Cr(VI) ions are still challenging and desperately required in the perspective of health issues and environmental protection. Lately, carbonaceous hydride materials have attracted immense attention because of their particular aspects such as monitoring in real-time, high sensitivity and quick response. Thus, the luminescent hydride NPs have been synthesized as sensors for the detection of Cr(VI) ions^9^. Recently, we have reported the synthesis and characterization of a novel highly luminescent PANI/Ag (AMPSA)/ GO QDs NC^13^. It was proposed that the interaction between Ag (AMPSA) NPs and PANI by the functional groups on the surface to facilitate the adsorption process. GO QDs are interacted strongly with PANI/Ag (AMPSA) NC via electrostatic interaction. Moreover, there are several studies reported the interaction mechanisms of graphene or GO within the hydride materials^14–17^.

The objective of this work is to use the high fluorescence property of this NC to determine the linearity, sensitivity, dynamic range and the detection limit of Cr(VI) in the range from 0.01 mg/L to 7.5 mg/L. The selectivity and interference of PANI/Ag (AMPSA)/GO QDs NC in presence of different metals ions are investigated. The influence of pH on the PL intensity and on the quenching efficiency of PANI/Ag (AMPSA)/ GO QDs NC is studied in the range of 2–10. The possible mechanisms for the interaction between PANI/Ag (AMPSA)/GO QDs NC and Cr(VI) also discussed.

## Materials and methods

### Preparation and characterization techniques

Ag (AMPSA) NPs were synthesized by the chemical reduction of silver nitrate using sodium borohydride as a reducing agent. GO QDs were prepared by glucose pyrolysis and dodecylbenzene sulfonic acid doped PANI was prepared by chemical oxidative polymerization method, respectively. In addition, PANI/Ag (AMPSA)/GO QDs NC was prepared by in situ oxidative polymerization of aniline in presence of the nanoparticles. All details regarding the synthesis and characterization of PANI/Ag (AMPSA)/ GO QDs NC and its related materials in the present study can be found in our recent publication^13^.

### Detection of Cr(VI) using luminescence spectra

A stock solution of 100 mg/L of Cr(VI) was prepared by dissolving potassium dichromate (0.0283 g) in 100 mL of deionized water. The detection of Cr(VI) was performed at room temperature and pH 6. The sensing experiments of Cr(VI) were carried out by monitoring the PL behavior of the PANI/Ag (AMPSA)/GO QDs NC solution. Briefly, series of concentrations of Cr(VI) (0–7.5 mg/L) were prepared. The following was added sequentially to a Falcon 15 mL conical centrifuge tubes: 2 mL of deionized water followed by the addition of a certain concentration of Cr(VI) ions. The pH of this solution was adjusted to 6 using 0.1 M of NaOH and HCl then diluted with deionized water to a volume of 3 mL. Finally, a volume of 100 μL of PANI/Ag (AMPSA)/GO QDs NC solution was mixed with Cr(VI) solution. The solution was mixed using a vortex mixer and was incubated for 10 min. The PL spectra of the resulting solutions were recorded by the excitation wavelength at 270 nm (λex = 270 nm) and the emission data were collected in the range of 290–500 nm. The maximum fluorescence intensity obtained at 348 nm was used for the quantification of Cr(VI). For analytical parameters optimization, the slope of calibration curve was used based on Eq. (1)^18^:

1 $$(F_{0} - F)/F_{0} = a C_{{Cr\left( {VI} \right)}} + b$$

where F_0_ and F represent the PL intensities of PANI/Ag (AMPSA)/GO QDs in the absence and presence of Cr(VI) ions, respectively and C_Cr(VI)_ is the concentration of chromium (mg/L). The slope and the intercept of the calibration curve are represented by a and b, respectively. The detection limit (DL) can be estimated from the equation of DL = 3SD/slope, where SD is the standard deviation of the calibration curve^19^. The quenching efficiency (QE) was calculated using Eq. (2)^20^:

2 $$QE = (F_{0} - F)/F_{0}$$

For optimization of experimental detection conditions, the effects of different parameters such as pH and the reaction time between PANI/Ag (ANPSA)/GO QDs NC and Cr(VI) were investigated. In addition, selectivity of PANI/Ag (AMPSA)/GO QDs toward Cr(VI) and the potential of interference with other ions including Fe(III), Mg(II), Pb(II), Cu(II), Zn(II), K(I), Cd(II), Ni(II), Cr(III), Na(I) and Al(III) were also examined.

### Detection of Cr(VI) in water samples

The feasibility of the proposed method for practical applications was investigated in the determination of Cr(VI) in two water samples (tap water and raw water obtained from drinking water canal, Alexandria, Egypt). The raw water was filtered to remove the impurities. All the pH of the sample was adjusted as 6. For validation purpose, the two water samples were analyzed by applying the standard colorimetric method using 1,5-diphenylcarbazide (DPC)^21^. Then, adding 5 mg/L of Cr(VI) into the samples and measuring the Cr(VI) concentrations again. The recovery percentages were also determined.

## Results and discussion

### Characterization

The structure, crystallinity and morphological properties of this NC were characterized and discussed and presented in other published work^13^.

### Surface charges of Ag (AMPSA) NPs, GO QDs and PANI/Ag (AMPSA)/GO QDs

Zeta potential measurement is applied to determine the surface charge and consequently the stability of colloidal Ag (AMPSA) NPs, GO QDs and PANI/Ag (AMPSA)/GO QDs s as shown in Table 1. It is observed that the prepared nanomaterials have a negative surface charge. In addition, the zeta potential of the stable aqueous PANI/Ag (AMPSA)/GO QDs NC is − 30.3 mV and it is the most stable among the other nanomaterials. Colloids with zeta potential higher than 30 mV (negative or positive) are stable^22^. According to the results obtained from Table 1, the stability of the synthesized materials colloidal decreases in the order of PANI/Ag (AMPSA)/GO QDs > Ag (AMPSA) NPs > GO QDs.

**Table 1:** Zeta potential of the as-prepared aqueous NPs and NC.

Specimen	Zeta potential (mV)
Ag (AMPSA) NPs	− 26.9
GO QDs	− 13.9
PANI/Ag (AMPSA)/GO QDs	− 30.3

### pH dependent fluorescence spectra of PANI/Ag (AMPSA)/GO QD NC

**Figure 1:**
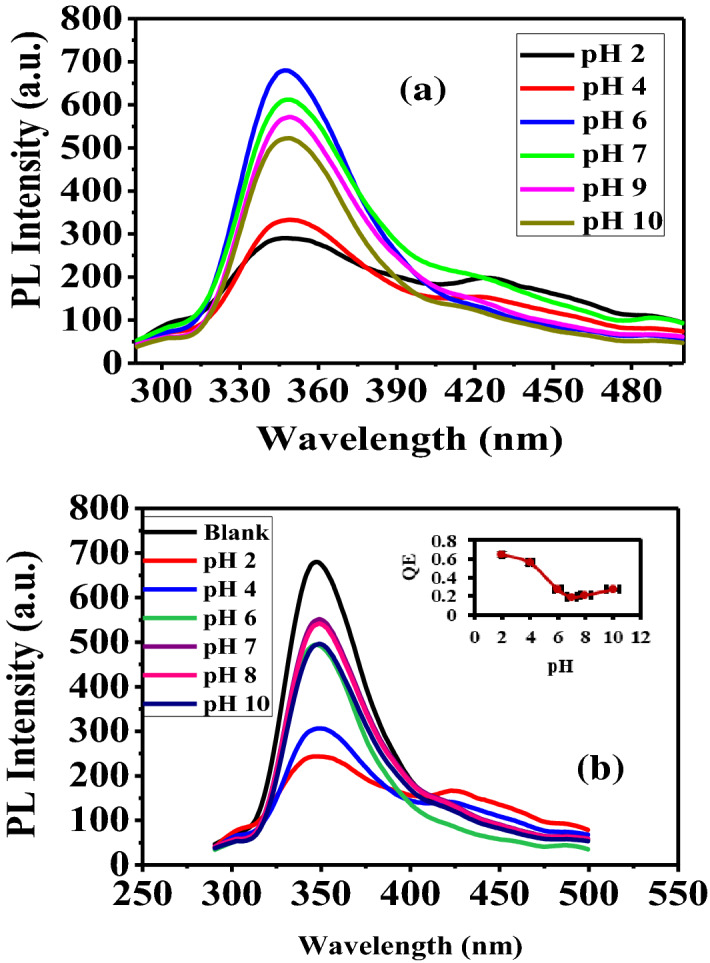
PL spectra of aqueous PANI/Ag (AMPSA)/GO QDs NC (**a**) in the absence and (**b**) in the presence of 0.5 mg/L Cr(VI) at different pHs, inset: QE versus pH at λex = 270 nm.

The effect of pH on both of the PL intensity and the quenching efficiency (QE) of PANI/Ag (AMPSA)/GO QDs in the absence and presence of 0.5 mg/L Cr(VI) solution is investigated and carried out in the pH range from 2 to 10 as depicted in Fig. 1a,b. The PL intensity of PANI/Ag (AMPSA)/GO QD is improved in the range of pH from 2 to 6. However, photoluminescence spectra is gradually declined again with further rising the pH from 6 to 10 as presented in Fig. 1a. This is can be explained based on the protonation and deprotonation of the amino groups at the PANI surface. It is proposed that the benzenoid rings are responsible for the fluorescence while at low pH the PANI is protonated and the conductive (quinoid rings) regions act as a fluorescence quencher^23^. Moreover, at low pH the aggregation of PANI/Ag (AMPSA)/GO QDs is manifested by the action of intramolecular hydrogen bond of the carboxylic acid moieties existed onto the surface of the PANI/Ag (AMPSA)/GO QDs^24,25^. On the other hand, PANI/Ag (AMPSA)/GO QDs is exhibited a new fluorescence band in the visible region (427 nm) at pH 2 as a result of the formation of ground state species by electron donor–acceptor interaction between emeraldine salt and GO QDs or charge transfer interaction within the PANI-GO QDs^26^. Furthermore, at higher pH (7–10) deprotonation of amino groups of the PANI and carboxylic groups of GO QDs is generated negative charges on the surface and forms anionic double layers. This double layer disturbs the fluorescence spectra of the NC^25^.

Figure 1b illustrates the effect of pH on the PL intensity and the QE of PANI/Ag (AMPSA)/GO QDs NC in the presence of 0.5 mg/L Cr(VI). It is observed that the dependence of PL on the pH has the same trend in the absence of Cr(VI). However, the highest fluorescence is obtained at pH 7. The QE value of Cr(VI) is about 0.2–0.3 at pH of 6–10 and about 0.6–0.5 at the pH of 2–4. The higher QE at low pH is attributed to the low fluorescence intensity of the PANI/Ag (AMPSA)/GO QDs. The excitation energy exceeded the energy band gap is absorbed by PANI/Ag (AMPSA)/GO QDs and consequently the electrons are promoted from the valence band to the conduction band. These electrons are relaxed and returned again to their ground states by emission photons with longer wavelength^27^. It is found that the presence of Cr(VI) ions produces a quenching in the fluorescence of the PANI/Ag (AMPSA)/GO QDs.

**Figure 2:**
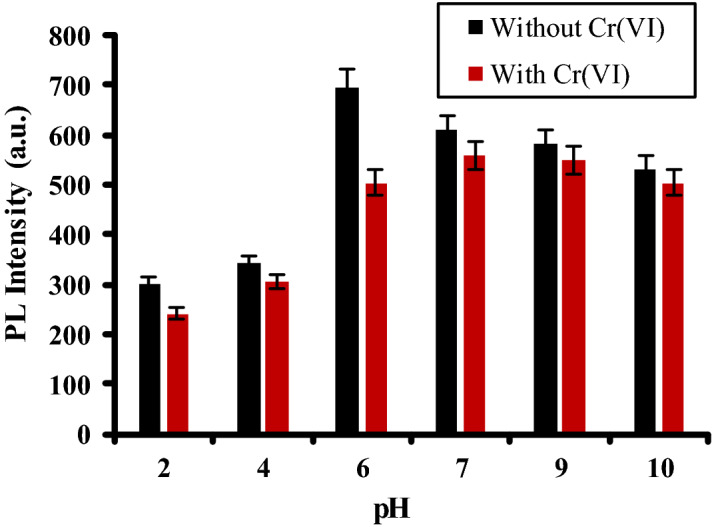
PL intensity of aqueous PANI/Ag (AMPSA)/GO QDs NC without and with 0.5 mg/L Cr(VI) versus pH at λex = 270 nm.

Figure 2 compares between the PL intensity of PANI/Ag (AMPSA)/GO QDs in absence and presence of 0.5 mg/L Cr(VI) at different pHs. The maximum PL intensity of PANI/Ag (AMPSA)/GO QDs without Cr(VI) is appeared at pH 6. It is noted that at this pH, QE of Cr(VI) towards PANI/Ag (AMPSA)/GO QDs is optimal (about 0.3). Therefore, pH 6 is chosen as the pH value for the detection of the Cr(VI) ions in the aqueous solution.

### Interaction mechanism between PANI/Ag (AMPSA)/GO QDs and Cr(VI)

**Figure 3:**
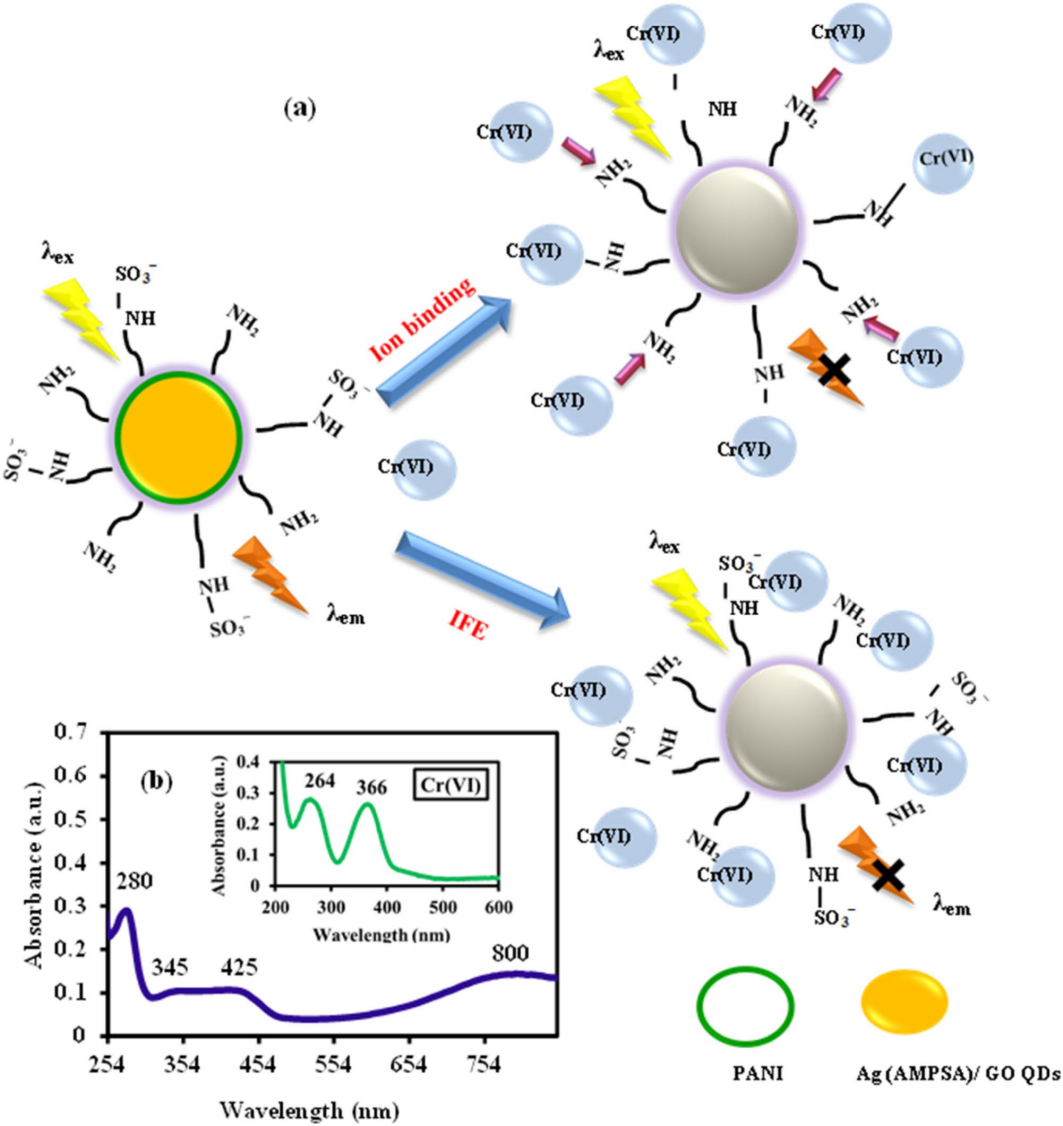
(**a**) Schematic illustration of the quenching processes of PANI/Ag (AMPSA)/GO QDs NC by Cr(VI) ions, λem is the emission wavelength and (**b**) UV–Vis spectra of PANI/Ag (AMPSA)/GO QDs NC and 5 mg/L Cr(VI) (the inset).

Generally, the luminance quenching mechanisms by ions exchange of metal ions^28^, ligands competition and binding^29^, electron and energy transfer^30,31^, and inner filter process^32^ are possible to occur. The quenching processes of PANI/Ag (AMPSA)/GO QDs PL spectra by Cr(VI) ions are illustrated in Fig. 3a. Inner filter effect requests an overlap between the absorption spectrum of the absorber, the excitation spectrum and emission band of the QDs^33^. As shown in the inset in Fig. 3b, the UV–vis spectrum of Cr(VI) displays absorption peaks at 264 and 366 nm. Figure 3b depicts that PANI/Ag (AMPSA)/GO QDs NC exhibits high absorption band at 280 nm, two shoulders at 345 and 425 nm, and a broad band at 800 nm, while strong emission peak at 348 nm. The spectral overlap between the absorption peaks of Cr(VI) and the absorption and emission spectra of PANI/Ag (AMPSA)/GO QDs indicates the presence of filter effect. Moreover, the absorption spectra of Cr(VI) can be shielded part of the radiation for the excitation of the PANI/Ag (AMPSA)/GO QDs, as well as the Cr(VI) ions can absorb partially the emitted light by the PANI/Ag (AMPSA)/GO QDs. This supports also the inner filter effect between the Cr(VI) and the PANI/Ag (AMPSA)/GO QDs, and consequently reduces the intensity of fluorescence spectra^18,20^.

The zeta potential of the PANI/Ag (AMPSA)/GO QDs NC is demonstrated to be − 30.3 mV (Table 1), and Cr(VI) is a negatively charged as monovalent bichromate HCrO_4_^−^ under pH of 6–7^34^. Thus, a relatively long distance between PANI/Ag (AMPSA)/GO QDs and Cr(VI) is expected due to electrostatic repulsion^20^. However, it is observed that the maximum emission peak shifts from 348 to 336 nm (blue-shift) when PANI/Ag (AMPSA)/GO QDs interact with Cr(VI) ions as will be shown in Fig. 5a.

It has been reported that PANI could combine with Cr(VI) in an aqueous solution due to the ion exchange occurred between the dopant (− SO_3_^−^) and the negative Cr(VI) ions^21^. Also, the electron donating groups onto polyaniline structure (–NH_2_) of the PANI/Ag (AMPSA)/GO QDs can strongly interact with metal ions to form the stable metal complexes^35^. These results suggesting that the quenching mechanism is not related only to inner filter effect process, but also the ground state compounds formation and ion exchange^18,36^.

### Performance of PANI/Ag (AMPSA)/GO QDs for Cr(VI) detection

**Figure 4:**
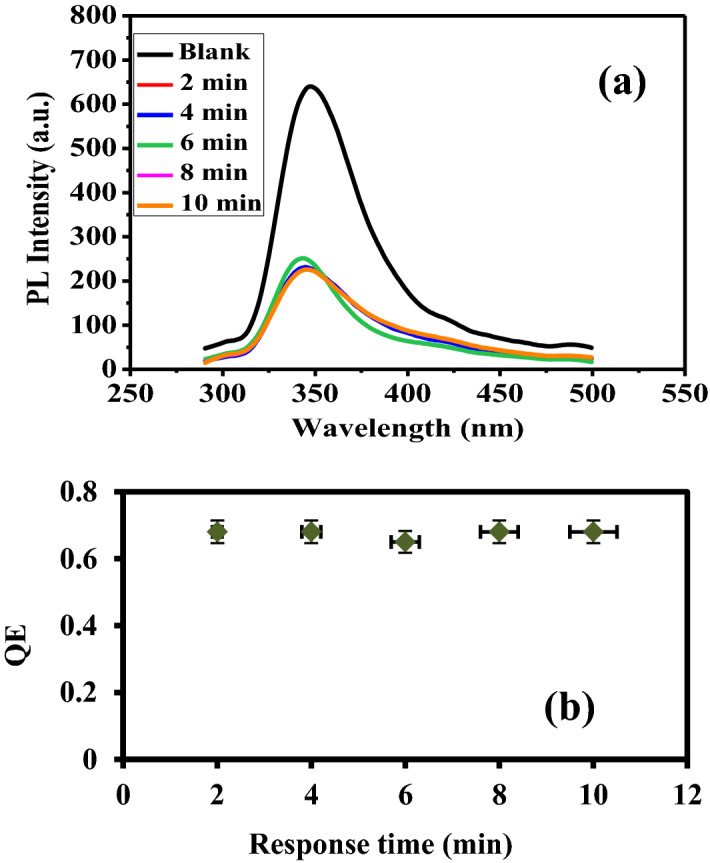
(**a**) PL intensity of PANI/Ag (AMPSA)/GO QDs in the presence of 5 mg/L Cr(VI) at different times and (**b**) QE versus response time at pH 6 and λex = 270 nm.

Upon addition of 5 mg/L of Cr(VI), the PL spectra and QE of PANI/Ag (AMPSA)/GO QDs NC are determined and plotted against the response time as demonstrated in Fig. 4a. The QE is reached equilibrium rapidly within approximately 2 min and then remained fixed (Fig. 4b). This reflects the high reactivity of PANI/Ag (AMPSA)/GO QDs towards Cr(VI) ions^37^. Therefore, the proposed fluorescent sensor for Cr(VI) not only achieves rapid detection, but also exhibits high stability.

**Figure 5:**
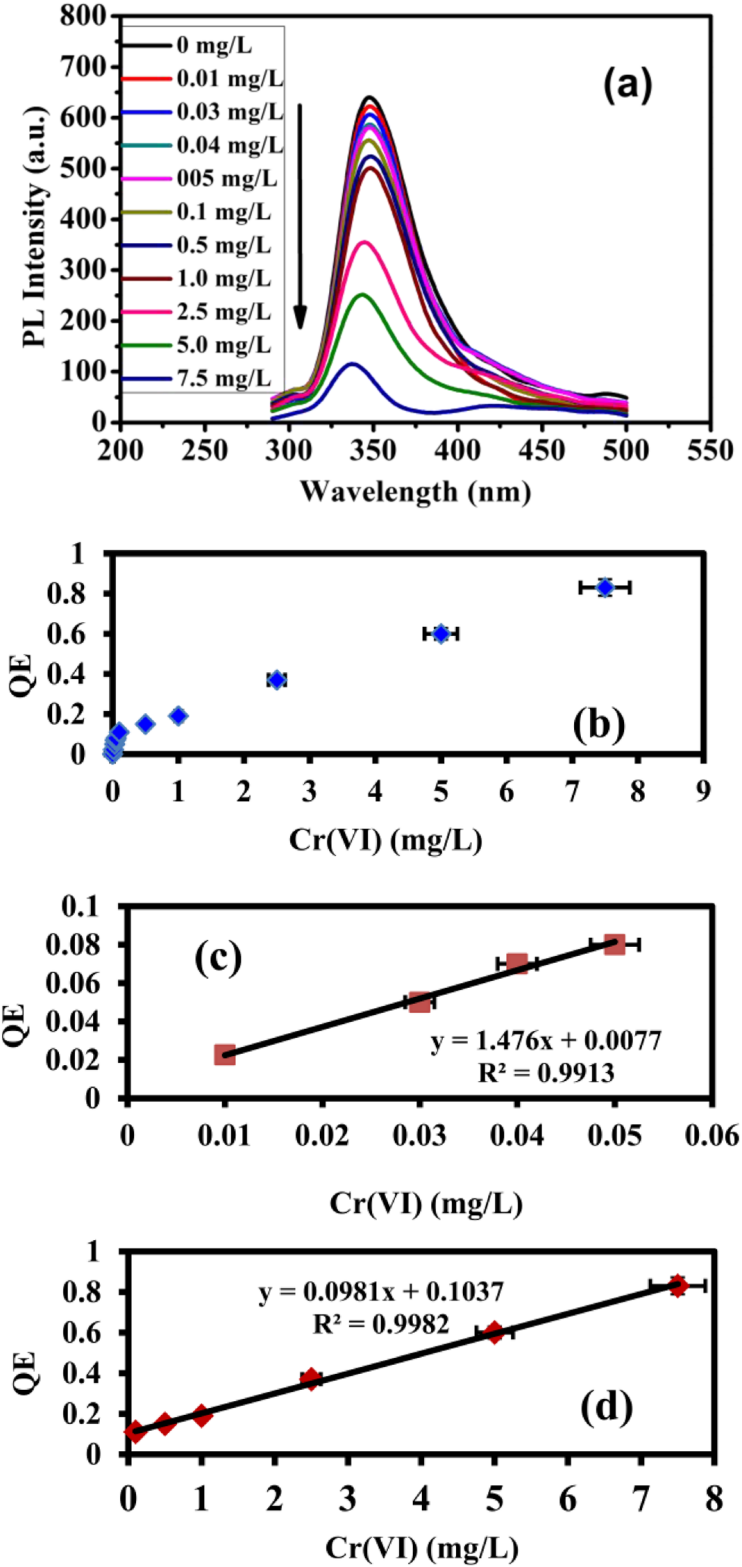
(**a**) PL spectra of PANI/Ag (AMPSA)/GO QDs with different concentrations of Cr(VI), (**b**) QE versus Cr(VI) concentration, (**c**) QE versus Cr(VI) concentration from 0.01 to 0.05 mg/L and (**d**) from 0. 1 to 7.5 mg/L at pH 6 and λex = 270 nm.

The linearity, sensitivity, dynamic range and the detection limit of PANI/Ag (AMPSA)/GO QDs sensor in the presence of series of concentrations of Cr(VI) (0–7.5 mg/L) are determined using PL property. It is observed that by increasing concentration of Cr(VI), the PL intensity gradually decreases and a blue-shift of about 12 nm is noted as shown in Fig. 5a. This shift is an indication of a complex formation between Cr(VI) and PANI/Ag (AMPSA)/GO QDs NC^18^. The PL quenching efficiency of PANI/Ag (AMPSA)/GO QDs is related to Cr(VI) concentration as presented in inset of Fig. 5b.

There are two linear regions (dynamic range) of the correlation in the Cr(VI) concentration range of 0.01–0.05 mg/L (R^2^ = 0.9913) and 0.1–7.5 mg/L (R^2^ = 0.9982) as shown in Fig. 5c,d, respectively. This indicates that the quantitative determination of Cr(VI) using PANI/Ag (AMPSA)/GO QDs sensor can be performed with different sensitivities (slopes of the calibration lines) of 1.476 and 0.0981 L/mg according to the range of detection. The detection limit obtained from the calibration line (Fig. 5c) is 0.0065 mg/L (~ 6 µg/L) calculated from 3SD/slope, where SD is the standard deviation and the slope of the calibration line. It is found that PANI/Ag (AMPSA)/GO QDs has a reasonable limit of detection for Cr(VI) ion in drinking water, which is lower than the maximum allowed limit of 50 μg/L set by the WHO. Therefore, PANI/Ag (AMPSA)/GO QDs based fluorescent sensor is sensitive enough to monitor Cr(VI) concentration in drinking water.

As listed in Table 2, the PANI/Ag (AMPSA)/GO QDs has low detection limit for Cr(VI) compared with other reported methods.

**Table 2:** Comparison of different methods for detecting Cr(VI).

Sensor	Method	Linear range	LOD (μg/L)	Ref
Ion-imprinted polymers/Mn-doped ZnS QDs	Fluorescent	20–1.0 mg/L	5.48	9
Carbon dots	Fluorescent	0.10–12 μg/mL	30.0	18
Glutathione/capped CdTe QDs	Fluorescent	0.01–1.00 μg/mL	8.0	38
GQD-modified membranes	Fluorescent	52–26 mg/L	9.9	39
Carbon dots	Fluorescent	0.1–1 mg/L	100.0	40
Phosphorus/carbon dots	Fluorescent	52–20.8 mg/L	12.5	41
Zinc oxide quantum dot/ Polyvinylpyrrolidone/ quantum carbon dots hydrogel composite	Fluorescent	0–67.7 μg/L	65.0	42
PANI/Ag (AMPSA)/GO QDs	Fluorescent	0.01–0.06 mg/L 0.1–7.5 mg/L	6.0	This work

The permissible limit of Cr(VI) in drinking water set by WHO = 50 μg/L.

### Stability, reproducibility and repeatability studies

The stability of PANI/Ag (AMPSA)/GO QDs was previously investigated^13^. The effect of ionic strength on the PL of PANI/Ag (AMPSA)/GO QDs NC was examined in the presence of different concentrations of NaCl. Results revealed that up to the solution of 500 mM NaCl has no significant effect on PL intensity due to the absence of interaction between the NC and NaCl. Also, the PL intensity of PANI/Ag (AMPSA)/GO QDs NC was measured after stored at 30 °C for different periods. PANI/Ag (AMPSA)/GO QDs illustrated a high resistance to photobleaching and the fluorescence intensity was declined by 16.3% after five weeks.

The reproducibility of the NC sensor is estimated by measuring the PL intensity of five different NC samples added to 5 mg/L Cr(VI) solutions prepared independently at pH 6. The relative standard deviation (RSD) is calculated to be 3.9%. Moreover, the repeatability of prepared NC is tested after 5 successive PL measurements of the same NC with 5 mg/L Cr(VI) solution at pH 6 and the RSD is found to be 2.4%. These results confirm that the PANI/Ag (AMPSA)/ GO QDs NC sensor is highly reproducible and can be applied for repeatable measurements.

### Selectivity and interferences of PANI/Ag (AMPSA)/GO QDs NC

**Figure 6:**
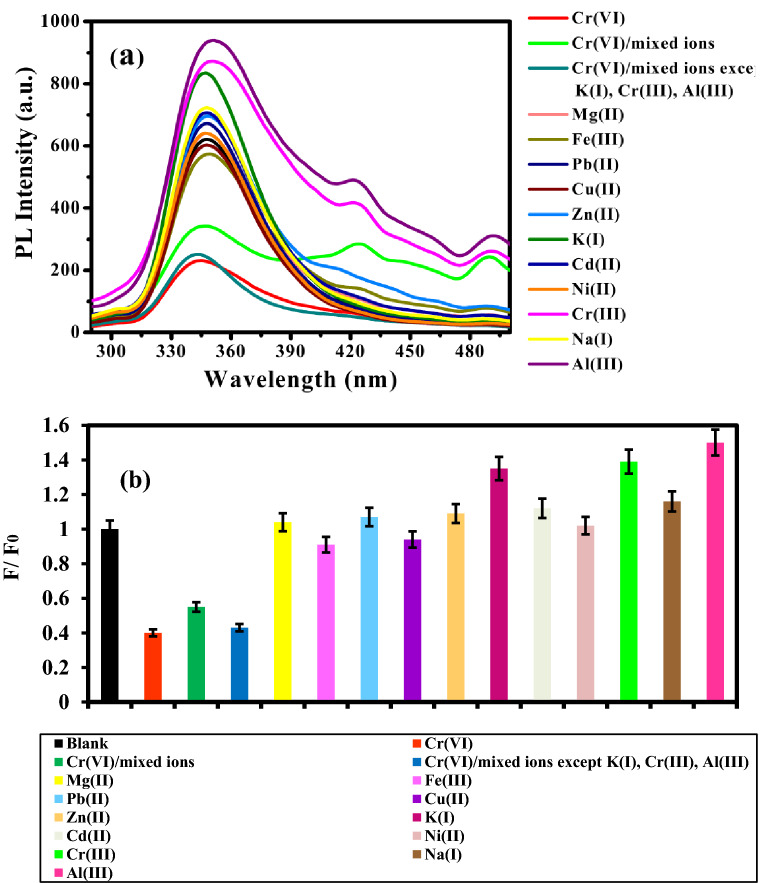
(**a**) PL intensity and (**b**) relative fluorescence intensity (F/F_0_) of PANI/Ag (AMPSA)/GO QDs versus 5 mg/L of different metal ions at pH 6, λex = 270 nm.

Selectivity is a very important parameter to evaluate the performance of the fluorescent sensor. The selectivity of PANI/Ag (AMPSA)/GO QDs NC toward Cr(VI) is investigated by testing the PL variation and the change in the relative fluorescence intensity (F/F_0_) as shown in Fig. 6a,b in the presence of fixed concentration of 5 mg/L of different metal ions including Cr(VI), Fe(III), Mg(II), Pb(II), Cu(II), Zn(II), K(I), Cd(II), Ni(II), Cr(III), Na(I) and Al(III) at pH 6 after incubation for 10 min. It is observed that the enhancement of the fluorescence intensity of PANI/Ag (AMPSA)/GO QDs NC in the presence of some metal ions (Al(III), Cr(III) and K(I)) is attributed to chelation enhanced fluorescence effect^43^. Upon the interaction of Cr(III), Al (III) and K(I) ions with the PANI/Ag (AMPSA)/GO QDs, it is observed that a fluorescence enhancement has occurred. This PL enhancement is not due to structural factor. Rather it is attributed to the blocking or stabilization of the nitrogen lone pair orbital by metal coordination^43,44^. Moreover, as presented in Fig. 6a, two new small peaks at around 422 and 489 nm are observed suggesting the formation of Cr(III), K(I) or Al(III)—ligand complex^45^. Consequently, PANI/Ag (AMPSA)/GO QDs have a potential application for monitoring Cr(III), Al(III) and K(I) in aqueous solutions.

It is also noted that Cr(VI) ions lead to a remarkable fluorescence quenching of PANI/Ag (AMPSA)/GO QDs whereas the other metal ions have a negligible quenching effect. To investigate the interference effect of different metals ions with Cr(VI), Cr(VI)/mixed ions sample was prepared by mixing 5 mg/L of each ions with the PANI/Ag (AMPSA)/GO QDs in presence of 5 mg/L Cr (VI). The small change in the relative fluorescence intensity (F/F_0_) of the PANI/Ag (AMPSA)/GO QDs from 0.4 to 0.55 is occurred due to interfering ions compared to the one of the sample contains only Cr(VI) in deionized water from 0.4 to 0.55 (Fig. 6b). We also carried out this experiment of Cr(VI)/mixed ions in the absence of Cr(III), Al(III) and K(I) as shown in Fig. 6a,b. The PL intensity of this mixture is almost similar to the PL of the solution contains only Cr(VI) ions. These results revealed that the Cr(VI)/mixed ions sample that contains all metal ions has lesser quenching than the sample involves only Cr(VI) due to the presence of Cr(III), Al(III) and K(I) ions which enhance the PL.

**Figure 7:**
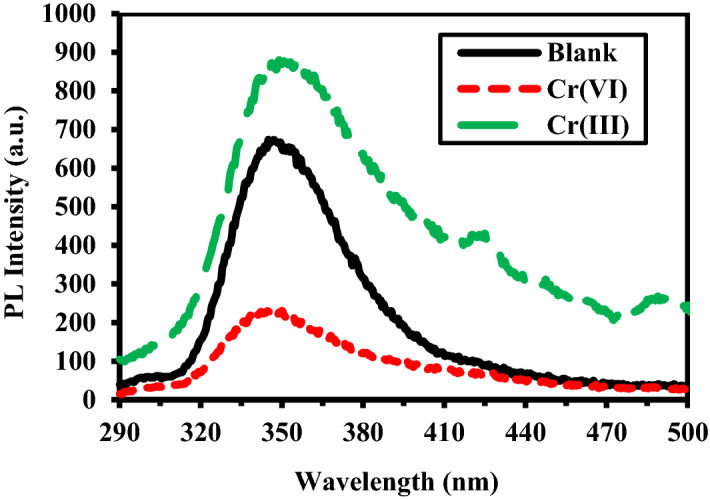
PL intensity of PANI/Ag (AMPSA)/GO QDs versus 5 mg/L of Cr(VI) and Cr(III) at pH 6, λex = 270 nm.

These results confirm that PANI/Ag (AMPSA)/GO QDs sensor is successfully able to discriminate between the two oxidation states of chromium ions (Fig. 7) where Cr(VI) quenches the PL and Cr(III) enhances the PL.

### Determination of Cr(VI) in water samples

**Figure 8:**
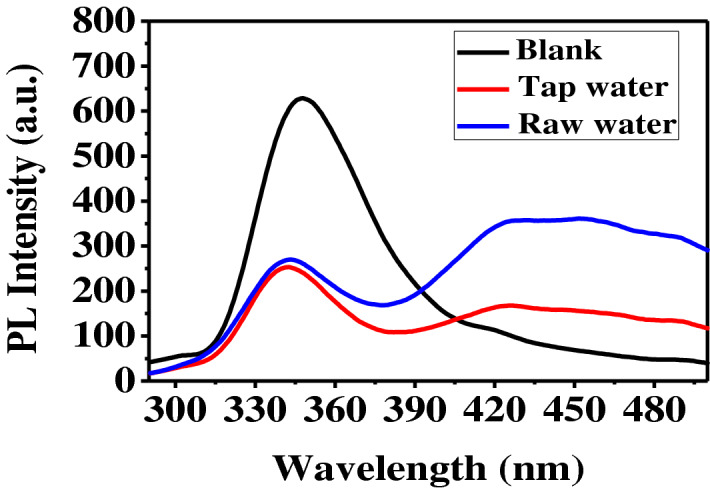
PL intensity of PANI/Ag (AMPSA)/GO QDs in local tap and raw water samples spiked with 5 mg/L Cr(VI) at pH 6, λex = 270 nm.

In order to validate this proposed nanocomposite and method, two local water samples spiked with 5 mg/L Cr(VI) was determined. The PL spectra of these two water samples are measured as shown in Fig. 8 from which the Cr(VI) concentrations were determined using the calibration curve in Fig. 5d. The Cr(VI) concentrations determined by the PANI/Ag (AMPSA)/GO QDs sensor are almost the same as those determined by the standard DFC method (5 mg/L).

The recoveries are estimated by measuring the PL intensities of five different tap water and raw water samples, each sample includes 5 mg/L Cr(VI) prepared independently at pH 6. It is found that the recoveries are in the range of 95.3–99.2% as shown in Table 3. The RSD is calculated to be 4.1% and 4.2% for tap water and raw water. It can be concluded that PANI/Ag (AMPS)/GO QDs sensor has a promising potential for monitoring Cr(VI) in water.

**Table 3:** The measured and recovered of Cr(VI) ions in the two water samples.

Sample	Cr(VI) (mg/L)	Cr(VI) added (mg/L)	Cr(VI) found (mg/L)	Recovery (%)	RSD (%)
Tap water	N.D	5	4.96	99.2	4.1
Raw water	N.D	5	4.75	95.3	4.2

ND, not detected.

Recovery % = (concentration found/concentration spiked) × 100%.

## Conclusion

In summary, PANI/Ag (AMPSA)/GO QDs NC was used as a sensitive fluorescence quenching probe for detecting Cr(VI) successfully. This probe possessed a low detection limit of 6 µg/L which is lower than the WHO standard permitted limit for Cr(VI) in drinking water (50 µg/L). Furthermore, the PANI/Ag (AMPSA)/GO QDs NC sensor offered several advantages such as good selectivity, fast response time, and high stability, reproducibility and repeatability. The mechanism of quenching is possibly due to the synergistic effect of inner filter effect, the ground state compounds formation and ion exchange. Moreover, PANI/Ag (AMPSA)/GO QDs NC have a potential application for monitoring Cr(III), Al(III) and K(I) in aqueous solutions. The real applicability of the sensor was also tested by analyzing real water samples spiked with known concentration of Cr(VI) and satisfactory recovery of Cr(VI) was obtained. We believe that this proposed fluorescent sensor possesses high potentials in the recognition of Cr(VI) in real water samples.
